# Neural reward system reflects individual value comparison strategy in cost-benefit decisions

**DOI:** 10.1038/s42003-024-07210-5

**Published:** 2024-11-12

**Authors:** Zarah Le Houcq Corbi, Alexander Soutschek

**Affiliations:** Department for Psychology, Ludwig Maximilian University Munich, Munich, Germany

**Keywords:** Reward, Human behaviour

## Abstract

A core assumption in decision neuroscience is that individuals decide between options by comparing option-specific subjective reward values. Psychological accounts challenge this view and suggest that decisions are better explained by comparisons between choice attributes than by comparisons between option-specific values, casting doubts on the interpretation of activation in the neural reward system as subjective value signals. Here, we provide neuroimaging and pharmacological evidence that value-related neural activity follows the value comparison strategy employed by an individual on the psychological level. Neural model comparisons reveal that activation in the striatum, rather than generally reflecting attribute-wise or option-wise value comparisons, reflects the value comparison strategy that provides the best explanation for an individual’s choice behavior. Strikingly, manipulating activation in the dopaminergic reward system reveals that dopamine antagonism counteracts the engagement in an individual’s dominant value comparison strategy. Together, our findings provide evidence for the biological plausibility of psychological accounts of decision making and emphasize the importance of neural model comparisons to prevent misinterpretations of brain activation.

## Abstract (web summary)

Converging fMRI and pharmacological evidence suggests that the neural reward system encodes utility according to an agent’s preferred values comparison strategy.

## Introduction

Many decisions require humans to trade off the benefits against the costs of actions^1^. Examples of this range from choosing between immediately available smaller rewards versus more valuable long-term outcomes (intertemporal decisions) to deciding whether to benefit others via sacrificing one’s own resources (interpersonal decisions). Prominent accounts of cost-benefit decisions assume that humans decide between reward options by comparing the subjective values of the options^2–5^. In both intertemporal and interpersonal decisions, the subjective value of an option is thought to integrate reward magnitudes and costs according to hyperbolic functions: In intertemporal decisions, the subjective value of a reward declines hyperbolically with increasing time of reward delivery^6,7^, whereas the value of sharing money with others is hyperbolically reduced with increasing social distance of the benefitted person^8–10^. A large body of evidence suggests that the subjective values of delayed and shared rewards correlate with activation in the neural reward system, including the striatum and ventromedial prefrontal cortex (VMPFC)^4,9,11–14^. These neural findings are often interpreted as evidence for the biological plausibility of economic discount models of decision-making^4^. However, past studies did not investigate whether activation in the reward system might better be explained by alternative models of decision making.

Recent evidence from the psychological literature suggests that economic discount models may not correctly reflect how humans actually make cost–benefit decisions. Instead of comparing the discounted subjective values of reward options (option-wise comparisons), many humans may make intertemporal decisions by comparing the weighted differences in reward magnitudes and delay costs of the choice options (attribute-wise comparisons)^15–18^. Previous evidence suggests that individuals strongly vary in the extent to which they employ attribute-wise relative to option-wise decision strategies^15,16^, such that it might be misleading to assign the same utility function to all individuals in a given sample. However, if many agents do not rely on option-wise comparisons when making cost-benefit decisions, it seems implausible to assume that the neural reward system encodes option-wise value comparisons in these agents. This challenges the widely held assumption in decision neuroscience where activation in the reward system is thought to represent the subjective values of reward options^19,20^. Interestingly, indirect support for the hypothesis that the brain might encode attribute-wise utility comparisons stems from the dopamine literature, where dissociable striatal pathways (direct versus indirect path) are thought to process reward magnitudes and costs in decision-making^21–23^. This suggests that striatal activity may reflect the relative weight assigned to reward and cost attributes rather than an integrated subjective value signal.

Here, we tested the hypothesis (inspired by psychological findings for intertemporal decisions) that activation in the neural reward system is better explained by attribute-wise rather than by option-wise utility comparisons during individual and social decisions in two separate experiments. On the behavioral level, we expected to replicate previous findings that cost-benefit decisions are better explained by attribute-wise than by option-wise value comparisons (hypothesis 1). On the neural level, we tested in a neuroimaging study whether activation in the neural reward system (including VMPFC and striatum) is better explained by attribute-wise compared with option-wise decision strategies when making cost–benefit choices (hypothesis 2). Moreover, to provide evidence for a causal relationship between neural activation and value comparison strategies, we assessed whether pharmacological manipulation of dopaminergic activation changed the balance between attribute-wise and option-wise value comparisons (hypothesis 3). While the neuroimaging data provided no evidence that the reward system generally encoded attribute-wise or option-wise utility computations on the group level, we observed activation in the reward system to reflect value computations according to the decision strategy employed by individuals. In line with this, pharmacologically blocking activity in the dopaminergic reward system reduced the engagement in the decision strategy preferred by an individual. Together, this challenges the widespread assumption in decision neuroscience according to which the neural reward system encodes option-specific discounted subjective values and demonstrates that the reward system reflects the individually employed utility computation strategy.

## Results

**Fig. 1:**
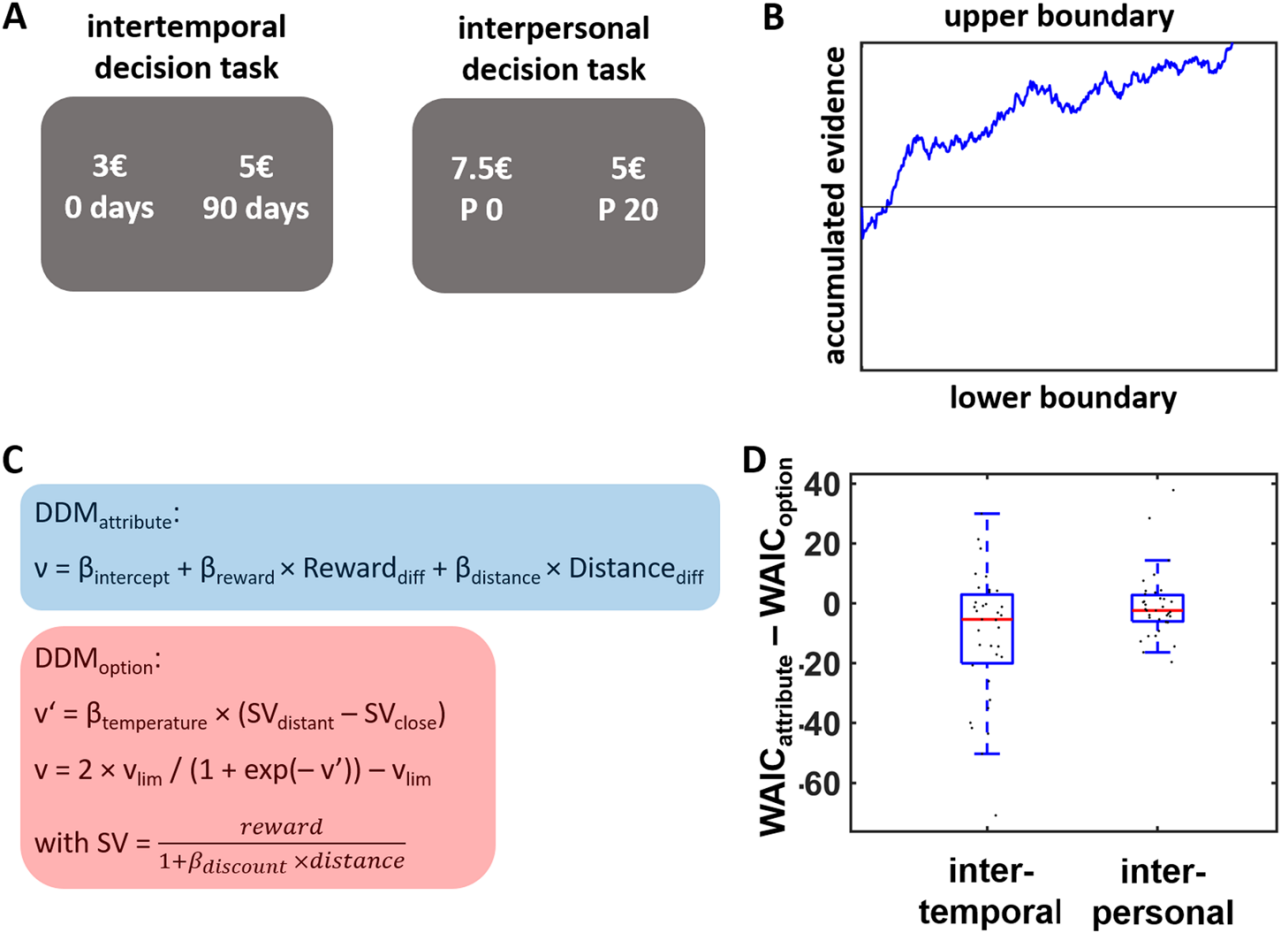
Task design and computational modeling approach. **A** Example trials for the intertemporal and interpersonal decision tasks. In the intertemporal decision task, participants made choices between temporally closer, smaller rewards (e.g., 3 euro today) and temporally distant, larger rewards (e.g., 5 euro in 90 days). The interpersonal decision task required choices between variable monetary rewards for a socially close person (including the participant, who was defined as social distance = 0; e.g., 7.5 euro for self) and 5 euro for both the socially close and a distant person (e.g., 5 euro for self and the person at social distance 20). **B** In both tasks, we fitted Bayesian drift-diffusion models (DDMs) to the behavioral data that assumed that decision-makers accumulate evidence with the velocity *ν* (drift rate) from a starting point *ζ* until the accumulated evidence reaches the decision boundary *α* for either the (temporally or socially) closer or more distant option (lower and upper boundaries, respectively). C In separate DDMs, we assumed that the drift rate reflects either attribute-wise or option-wise utility comparisons of choice alternatives. **D** Intertemporal choices were better explained (indicated by lower WAIC values) by DDM_attribute_ than by DDM_option_, whereas in the interpersonal decision task, we found no significant differences between DDM_attribute_ and DDM_option_. Boxes indicate inter-quartile range, red lines indicate the median, and black dots represent individual data points (*N* = 35 participants).

To test our hypotheses, 35 healthy young participants performed intertemporal and interpersonal decisions in the functional magnetic resonance imaging (fMRI) scanner. In the intertemporal decision task, participants decided between smaller-sooner and larger-later rewards (e.g., “3 euro in 0 days” versus “5 euro in 90 days”). In the interpersonal decision task, we first asked participants to imagine individuals at various social distances ranging on a scale from 1 (closest other) to 100 (a person they never met before). In the scanner, they then decided between a smaller reward for a close person (including themselves) and a larger reward split equally between the close other and a more distant other (e.g., “7.5 euro for self” versus “10 euro split equally between self and person at social distance 20”) (Fig. 1A).

To replicate and extend previous findings that choice behavior is better explained by attribute-wise rather than option-wise comparisons, we fitted drift-diffusion models (DDMs) to individual choice data^24^. We fitted the models on the individual rather than hierarchical level as this approach allows investigating individual variation in model fit between competing models. DDMs simulate decisions as an evidence accumulation process starting after a non-decision time τ until a decision boundary α is reached (where the upper boundary is associated with choices of larger and temporally or socially more distant rewards, the lower boundary with choices of smaller-closer rewards; Fig. 1B). The speed of the evidence accumulation process (drift rate *ν*) depends on the preference strength for one option over the other and was modeled based on either attribute-wise or option-wise value comparisons (Fig. 1C). More specifically, in the attribute-wise DDM (DDM_attribute_) the drift rate was given by the following equation:

1 $${{{\rm{\nu }}}}={{{{\rm{\beta }}}}}_{{{{\rm{intercept}}}}}+{{{{\rm{\beta }}}}}_{{{{\rm{reward}}}}}\times {{{{\rm{Reward}}}}}_{{{{\rm{diff}}}}}+{{{{\rm{\beta }}}}}_{{{{\rm{distance}}}}}\times {{{{\rm{Distance}}}}}_{{{{\rm{diff}}}}}$$

Reward_diff_ and Distance_diff_ indicate the differences in reward magnitude and distance, respectively, between the more distant and the closer option. *β*_reward_ and *β*_distance_ represent the individual weights assigned to differences in reward magnitude and distance, respectively, during evidence accumulation, whereas *β*_intercept_ is a participant-specific intercept reflecting the general speed of the evidence accumulation process (independently of the specific reward magnitudes and distances in a given trial) towards decision boundaries. We note that DDMs including the intercept parameter explained the data better (intertemporal: widely applicable information criterion (WAIC) = 206.1; interpersonal: WAIC = 225.0) than DDMs without intercept (intertemporal: WAIC = 223.3; interpersonal: WAIC = 235.7). Parameters were fit separately for the intertemporal and the interpersonal decision tasks. In analogy, in the option-wise DDM (DDM_option_), the drift rate was given by

2 $${\nu }^{\prime}={{{{\rm{\beta }}}}}_{{{{\rm{temperature}}}}}\times ({{{{\rm{SV}}}}}_{{{{\rm{distant}}}}}-{{{{\rm{SV}}}}}_{{{{\rm{close}}}}})$$

3 $${{{\rm{SV}}}}={{{\rm{reward}}}}\; {{{\rm{magnitude}}}}/(1+{{{{\rm{\beta }}}}}_{{{{\rm{discount}}}}}\times {{{\rm{distance}}}})$$

Here, *β*_discount_ represents a task- and participant-specific discount factor quantifying the degree to which temporal or social distance reduces the subjective value (SV) of delayed or shared rewards. The drift rate *v*’ was moreover passed through a sigmoid function with the upper and lower border *v*_lim_ (which also ensured that DDM_attribute_ and DDM_option_ entailed equal numbers of free parameters):

4 $${v}=2 \times {{v}}_{{{\mathrm{lim}}}}/(1+\exp (-{v}^{\prime}))-{v}_{{{\mathrm{lim}}}}$$

In line with previous evidence^25^, DDMs_option_ including this sigmoidal transformation (intertemporal: WAIC = 216.7; interpersonal: WAIC = 225.7) explained the data better than DDMs without this transformation (intertemporal: WAIC = 217.8; interpersonal: WAIC = 246.4). However, adding a sigmoidal transformation to DDM_attribute_ did not improve model fit (intertemporal with sigmoidal: WAIC = 208.6; intertemporal without sigmoidal: WAIC = 206.1; interpersonal with sigmoidal: WAIC = 227.7; interpersonal without sigmoidal: WAIC = 225.0). Model comparisons between DDM_attribute_ and DDM_option_ revealed that attribute-wise comparisons explained choice behavior better than option-wise comparisons in the intertemporal decision task, Wilcoxon signed-rank test between WAIC_attribute_ and WAIC_option_: *W* = 155, *p* = 0.008. In contrast, we observed no significant difference between WAIC_attribute_ and WAIC_option_ in the interpersonal decision task, Wilcoxon signed-rank test: *W* = 232, *p* = 0.20, thus providing only partial support for our first hypothesis. However, as indicated by Fig. 1D and consistent with previous findings^15,16^, in both tasks, there was substantial variation in the extent to which individuals employed attribute-wise relative to option-wise decision strategies.

**Fig. 2:**
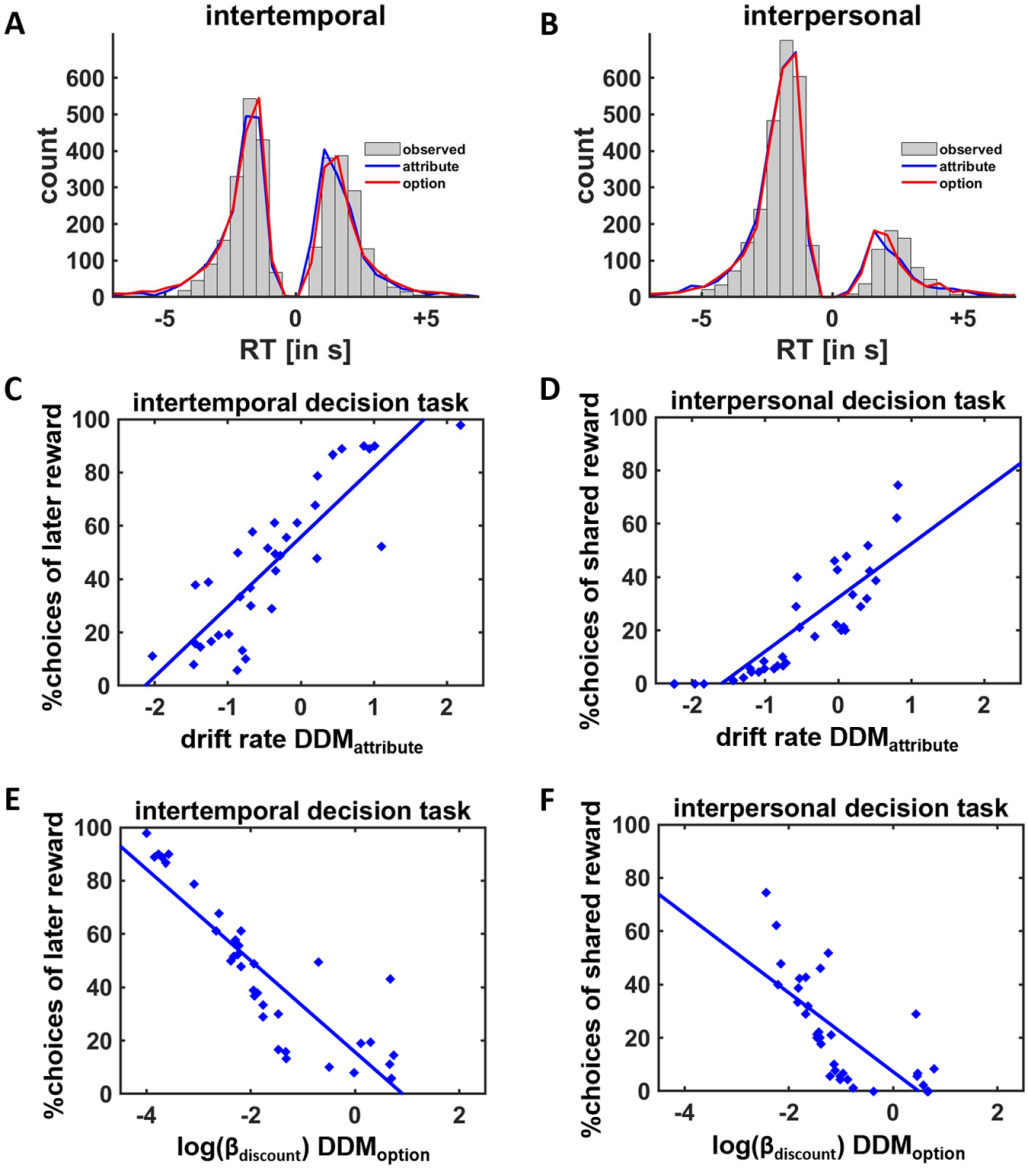
Behavioral results. **A**, **B** Posterior predictive checks revealed that decision times simulated based on parameter estimates from DDM_attribute_ and DDM_option_ showed strong overlap with the empirically observed decision times in both the intertemporal (**A**) and interpersonal (**B**) decision tasks. Negative and positive decision times indicate choices of the closer and more distant reward options, respectively. **C**–**F** Individual differences in the propensity to choose temporally or socially more distant over closer rewards were correlated with drift rates from **C**, **D** DDM_attribute_ as well as log-transformed discount parameters from **E**, **F** DDM_option_, suggesting that DDM_attribute_ and DDM_option_ captured essential aspects of time and social preferences (*N* = 35 participants).

Posterior predictive checks revealed that decision times simulated based on model parameters from both DDM_attribute_ and DDM_option_ closely matched the decision times observed in the intertemporal and interpersonal decision tasks (Fig. 2A, B). Moreover, DDM_attribute_ explained 83% of all intertemporal decisions and 81% of all interpersonal decisions, DDM_option_ correctly predicted 79% of all intertemporal decisions and 81% of all interpersonal decisions. This suggests that the models provide reasonable accounts of the empirical data, though they were still distinguishable as they predicted different choices in 24% of all intertemporal decision trials and in 20% of all interpersonal decision trials. To more thoroughly compare prediction accuracies between the models, we fitted the DDMs on 90% of the trials and tested the prediction accuracy in the remaining 10% of trials. This procedure was repeated 10 times to determine the mean prediction accuracy of the DDMs for each participant and decision task. In the intertemporal decision task, cross-validated prediction accuracy was significantly higher for DDM_attribute_ (80%) compared with DDM_option_ (76%), *t*(34) = 2.47, *p* = 0.02, whereas in the interpersonal decision task, prediction accuracy did not differ between DDM_attribute_ (80%) and DDM_option_ (81%), *t*(34) = 0.26, *p* = 0.81. This is consistent with the WAIC results according to which attribute-wise comparisons explain intertemporal decisions better than option-wise comparisons. Lastly, model recovery assessments showed that both intertemporal and interpersonal data simulated based on parameters from DDM_attribute_ could better be explained by DDM_attribute_ than DDM_option_, both *p* < 0.001, whereas data created based on DDM_option_ parameters were better explained by DDM_option_ than DDM_attribute_ in the intertemporal, *p* < 0.001, but not the interpersonal decision task, *p* = 0.80. On balance, this suggests that the models capture dissociable aspects of choice behavior.

Further sanity checks revealed that the sum of the weighted influences of rewards and costs on the drift rate in DDM_attribute_ (*β*_intercept_ + *β*_reward_ + *β*_distance_) significantly predicted mean choices of larger-later rewards, Kendall’s tau = 0.67, *p* < 0.001, and prosocial rewards, Kendall’s tau = 0.76, *p* < 0.001 (Fig. 2C, D). Likewise, mean choices were also negatively correlated with log-transformed hyperbolic discount parameters from DDM_option_ in the intertemporal, Kendall’s tau = -0.53, *p* < 0.001, and interpersonal decision task, Kendall’s tau = −0.61, *p* < 0.001 (Fig. 2E, F). Thus, our process models capture essential aspects of preferences for delayed and shared rewards and replicate previous findings according to which attribute-wise utility comparisons explain intertemporal choices better than option-wise comparisons^15,16,26^, and extend these findings to the social domain.

Note that in the current study, we cannot determine whether differences in model fit between DDM_attribute_ and DDM_option_ relate to participants’ information search behavior due to the lack of eye-tracking data. To address this issue, we fitted our DDMs to a previously published data set where eye tracking had been used to determine whether participants made attribute-wise or option-wise gaze transitions when searching for information (expressed via the Payne index^27^) in an intertemporal decision task^15^. The significant correlation between the Payne index and the WAIC_attribute_ minus WAIC_option_ difference score, *r* = 0.21, *p* = 0.03, suggests that our model-based index indeed captures variation in participants’ information search behavior.

**Fig. 3:**
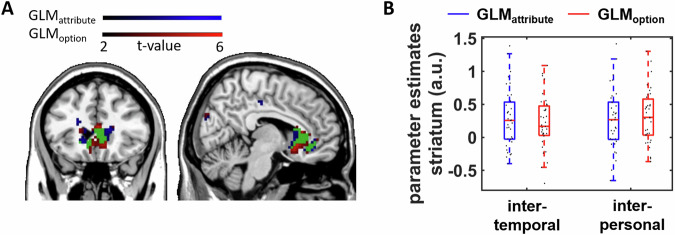
Neural correlates of attribute- and option-wise utility comparisons. **A** Value comparisons according to attribute-wise and option-wise decision strategies were encoded in overlapping regions (displayed in green) of the neural reward system, particularly in the striatum. **B** Separate analyses for the intertemporal and interpersonal decision tasks revealed that the striatum (ROI based on conjunction analysis) significantly encoded attribute- and option-wise utility comparisons in both individual and social decisions (*N* = 35 participants).

Next, we assessed whether the individual variation between attribute-wise and option-wise value comparisons on the psychological level is also reflected on the neural level. For this purpose, we computed two general linear models (GLMs) on the fMRI data: GLM_attribute_ assessed the neural correlates of the unsigned trial-varying drift rates (which reflect the strength of the preference for one option over the other) in intertemporal and interpersonal decisions as given by individual model parameters from DDM_attribute_. Analogically, GLM_option_ modeled preference strengths between reward options via individual parameters from DDM_option_. Unsigned drift rates in GLM_attribute_ and GLM_option_ showed mean correlations (correlations were computed separately for each participant) of *r* = 0.66 in the intertemporal decision task and *r* = 0.47 in the interpersonal decision task. Because in DDM_option_ drift rates depended on hyperbolically discounted subjective reward values, GLM_option_ is equivalent to previous studies investigating the neural correlates of value differences based on discounted subjective values^4,9,14,28^. Consistent with these previous findings, unsigned differences between discounted reward values in GLM_option_ were encoded in the striatum as part of the neural reward system (with the cluster extending into VMPFC), peak coordinates: *x* = 2, *y* = 12, *z* = −9, whole-brain family-wise error (FWE) corrected at the cluster level, *p* = 0.04 (Supplementary Table 1). Importantly, we found the striatum to be active also when modeling value differences between reward options via the drift rates from the attribute-wise DDMs; peak coordinates: *x* = −4, *y* = 15, *z* = 15, whole-brain FWE corrected at cluster level, *p* < 0.001 (Supplementary Table 2). A conjunction analysis revealed that the striatum clusters related to attribute-wise and option-wise utility computations strongly overlapped (Fig. 3A). When we extracted parameter estimates from this overlapping cluster separately for the interpersonal and intertemporal decision task, striatal activation in both tasks correlated with attribute-wise, both *t*(34) > 3.59, both *p* < 0.003, and option-wise utility computations, both *t*(34) > 3.34, both *p* < 0.002 (Fig. 3B). This result was robust to extracting parameter estimates from a meta-analysis-based striatum ROI, all *t*(34) > 2.74, all *p* < 0.02, Bonferroni-corrected, whereas a VMPFC ROI from this meta-analysis revealed significant effects only for attribute-wise comparisons in the intertemporal decision task, *t*(34) = 3.71, *p* = 0.002, Bonferroni-corrected, all other effects *t*(34) < 1.19, *p* > 0.48. We, therefore, restricted our further analyses to the striatum ROIs. In both tasks there was no evidence that activity within the striatum ROIs significantly differed between GLM_attribute_ and GLM_option_, all *p* > 0.28. Thus, the striatum correlated with the strength of individual and social preferences according to both option-wise and attribute-wise value computations.

As a control analysis, we assessed whether value-related activation in the neural reward system might better be explained by models fitted to binary choice data rather than DDMs (which are fitted to both choices and decision times). We, therefore, estimated parameters for the attribute-wise and option-wise models by fitting them to binary choices (using the dbern function in JAGS), following previous procedures in imaging research^4,9,28^. Attribute-wise models explained the binary choice data better (indicated by lower WAIC values) than option-wise models both in the intertemporal and interpersonal decision tasks, both *p* < 0.001. To compare how well the two modeling approaches explained the neural data, we re-computed GLM_attribute_ and GLM_option_ based on parameters from the choice-only models. Value-related activation in the striatum and VMPFC ROIs was slightly better explained (i.e., lower BIC values) by the DDM-based (GLM_attribute_: BIC_VMPFC_ = 7516.2, BIC_striatum_ = 7317.0; GLM_option_: BIC_VMPFC_ = 7516.9, BIC_striatum_ = 7318.1) than by the binary choice-based imaging analyses (GLM_attribute_: BIC_VMPFC_ = 7516.9, BIC_striatum_ = 7317.2; GLM_option_: BIC_VMPFC_ = 7518.4, BIC_striatum_ = 7318.1). This justifies our approach of analyzing neural value correlates based on DDM parameters.

**Fig. 4:**
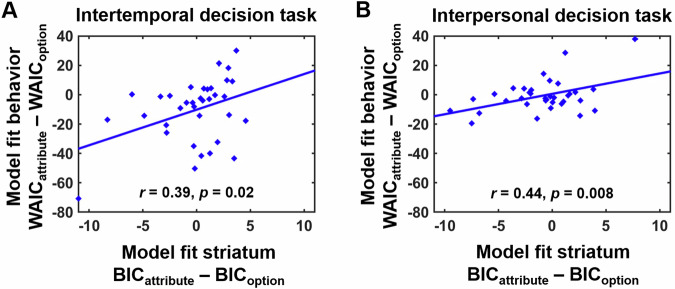
Model comparisons at the neural level. While activation in the striatum showed no significant differences in model fit between attribute-wise (GLM_attribute_) and option-wise (GLM_option_) utility comparisons on the group level (assessed with the Bayesian information criterion, BIC), model comparisons at the neural level significantly correlated with differences in model fit at the behavioral level (widely applicable information criterion, WAIC) both in the **A** intertemporal and the **B** interpersonal decision task (*N* = 35 participants). Thus, the neural reward system reflected the decision strategy employed by an individual rather than encoding attribute-wise or option-wise value comparisons per se.

Although on the group level, both attribute-wise and option-wise value comparisons correlated with striatal activity, it seems implausible that one brain region indeed encodes both value comparison strategies simultaneously. The question of whether striatal value signals are better explained by attribute-wise or option-wise comparisons can be answered via model comparisons on the neural level. When comparing model fits between GLM_attribute_ and GLM_option_ with the MACS toolbox^29^, Bayesian information criteria (BIC) values extracted from the striatum ROI based on overlapping value representations did not significantly differ between GLM_attribute_ and GLM_option_ both in the intertemporal, *t*(34) = 0.32, *p* = 0.73, and the interpersonal decision task, *t*(34) = 1.57, *p* = 0.12. This does not support our second hypothesis that the neural reward system more strongly represents attribute-wise than option-wise value comparisons. However, given the substantial inter-individual variation in the extent to which participants’ choice behavior was better explained by either DDM_attribute_ or DDM_option_, we assessed whether model comparisons at the neural level (BIC_attribute_ minus BIC_option_ from the neural analyses) reflected the employed decision strategy in behavior (WAIC_attribute_ minus WAIC_option_ from the DDM analyses). In fact, in the data-driven striatum ROI we observed significant correlations between model fits on the behavioral and the neural level both in the intertemporal, *r* = 0.39, *p* = 0.02, and the interpersonal decision task, *r* = 0.44, *p* = 0.008 (Fig. 4). These correlations were robust to extracting neural BICs from the alternative striatum ROI based on a meta-analysis on neural value coding^19^, intertemporal: *r* = 0.40, *p* = 0.02, interpersonal: *r* = 0.39, *p* = 0.02. Taken together, rather than generally encoding attribute-wise or option-wise value comparisons, the striatum appears to reflect the decision strategy employed by an individual.

### Dopamine antagonism inhibits dominant value comparison strategy

As the striatum is the target of the dopaminergic system, the findings for the striatum reported above suggest that dopaminergic activity might encode value comparisons according to the strategy pre-dominantly employed by an individual rather than generally representing attribute-wise or option-wise utility comparisons. In keeping with this, pharmacological manipulations of dopamine levels were found to change the weights assigned to rewards in attribute-wise utility comparisons^21,26^, whereas other studies reported dopamine effects on option-specific hyperbolic discounting^25,30,31^. To test whether dopamine is sensitive to the value comparison strategy employed by an individual, we re-analyzed a previously published data set assessing the impact of the D2 antagonist amisulpride (relative to placebo) on intertemporal and interpersonal decision-making in 56 healthy young volunteers (within-subject design)^12,26^. Using the same modeling approach as in the imaging study, we fitted DDM_attribute_ and DDM_option_ to each participant’s intertemporal and interpersonal decision data, separately for the amisulpride and placebo conditions. To measure the influence of amisulpride on whether decision data are better explained by attribute-wise (WAIC_attribute_) versus option-wise (WAIC_option_) value processing, we subtracted the differences in model fit (WAIC_attribute_–WAIC_option_) under amisulpride from the differences in model fit under placebo (WAIC_diff_amisulpride_placebo_ = (WAIC_attribute_–WAIC_option_)_amisulpride_–(WAIC_attribute_–WAIC_option_)_placebo_), separately for each task. We then regressed individual differences in drug effects on value computation (WAIC_diff_amisulpride_placebo_) on predictors for Task (intertemporal vs. interpersonal), the dominant decision strategy under placebo (WAIC_diff_placebo_ = (WAIC_attribute_–WAIC_option_)_placebo_), Session (amisulpride administered in session 1 vs. 2), and all interaction effects. The predictor Session allowed us to dissociate drug effects from a potential regression to the mean (extreme values for WAIC_diff_ in the first session might tend towards the mean in the second session), whereas the predictor WAIC_diff_placebo_ indicated whether the influence of amisulpride depended on the dominant value processing strategy (i.e., WAIC_attribute_–WAIC_option_) under placebo. A positive relationship between WAIC_diff_placebo_ and WAIC_diff_amisulpride_placebo_ would indicate that dopamine receptor blockade further strengthens an individuals’ dominant value comparison strategy, while a negative relationship would suggest that reducing dopaminergic activity attenuates the dominant value computation mode.

**Fig. 5:**
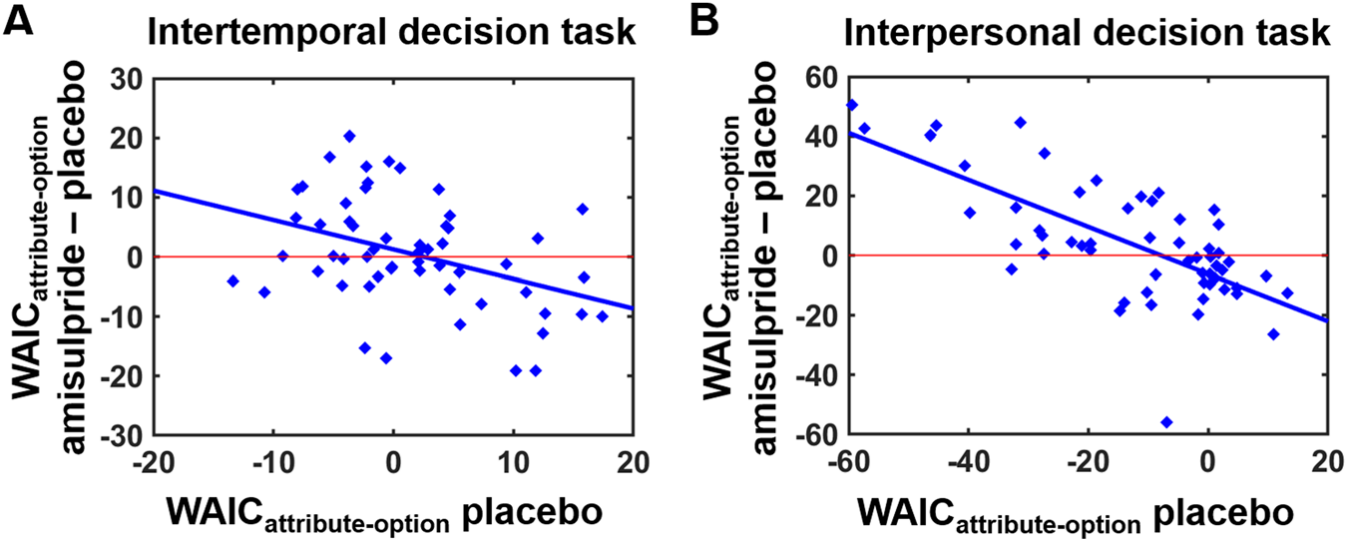
Influence of dopamine on value comparisons. Reducing dopaminergic activity with amisulpride (compared with placebo) attenuated the engagement in an individual’s dominant value comparison strategy both in the **A** intertemporal and the **B** interpersonal decision task (*N* = 56 participants). In interpersonal decisions, D2R inhibition is also generally promoted option-wise over attribute-wise value comparisons.

The main effect of the Task, beta = 3.90, *t*(81) = 2.99, *p* = 0.004, indicated that amisulpride (relative to placebo) promoted option-wise over attribute-wise value comparisons more strongly in the interpersonal than in the intertemporal decision task. Importantly, we found that the strength of the amisulpride effects on the balance between attribute-wise and option-wise value computations depended on the dominant decision strategy under placebo, the main effect of WAIC_diff_placebo_: beta = −9.61, *t*(104) = 6.11, *p* < 0.001 (Supplementary Table 3). This is consistent with the hypothesis that dopamine encodes value comparisons according to the strategy employed by an individual. Separate post-hoc analyses for the intertemporal and interpersonal decision task revealed significant main effects of WAIC_diff_placebo_ both in the intertemporal, beta = −4.07, *t*(54) = 3.88, *p* < 0.001, and the interpersonal decision task, beta = −11.65, *t*(54) = 7.06, *p* < 0.001 (Fig. 5). We observed no significant interaction between Session and WAIC_diff_placebo_ under placebo, beta = 1.03, *t*(104) = 0.65, *p* = 0.51, providing no evidence for a regression to the mean (we note, however, that for formally ruling out any influences of a regression to the mean it would have been necessary to add a second placebo session to our experimental design). Lastly, to rule out that the observed effects of amisulpride on attribute- versus option-wise value processing can be explained by reduced choice consistency, we assessed drug effects on inverse temperature parameters from the option-wise DDMs. While in the interpersonal decision task inverse temperature parameters did not differ between amisulpride and placebo, *t*(55) = 0.88, *p* = 0.38, in the intertemporal decision task, choice consistency was increased (rather than reduced) under amisulpride, *t*(55) = 2.57, *p* = 0.01. This does not support the alternative explanation according to which the drug effects on value processing might be driven by enhanced decision noise. Taken together, the blockade of dopaminergic D2 receptors impaired value computations according to the individually preferred decision strategy, mirroring the results of the model comparisons for the neural reward system in the imaging study.

## Discussion

Numerous studies on the neural basis of decision-making are based on the (often implicit) assumption that people decide between different options by comparing discounted subjective reward values^4,9,14,28,32,33^. Here, we show that it may be too simplistic to assume the reward system to implement the same utility function in all individuals and contexts. Instead, the dopaminergic reward system (and particularly the striatum) seems to reflect the decision strategy employed by a decision maker on the psychological level. While attribute-wise comparisons explained choice behavior better than option-wise comparisons only in the intertemporal (replicating previous findings^15,16^), but not interpersonal decision task (contrary to hypothesis 1), in both tasks we observed substantial variation in the value comparison strategy employed by decision makers. This heterogeneity in decision behavior could be explained by model comparisons at the neural level: While we observed no evidence that the neural reward system generally encodes economic preferences according to either attribute-wise or option-wise value comparisons (contrary to hypothesis 2), individual differences in model fit on the neural level reflected the dominant value comparison strategy on the psychological level. However, we observed such brain-behavior correlations only in the striatum, not in the VMPFC, suggesting that striatal activation might be more sensitive to the employed decision strategy. We also note that neural activation in the value coding ROIs was slightly better explained when computing value differences based on DDM parameters compared with purely choice-based models. We emphasize that these results were robust to different domains (individual versus social) of economic decision-making as well as to defining the reward system with a data-driven approach or based on a meta-analysis of value coding. Strikingly, the pharmacological data suggest that lowering dopaminergic activity attenuates the engagement in the value comparison strategy preferred by an individual, providing causal evidence that the dopaminergic reward system is sensitive to the decision strategy employed by an individual. While the observed correspondence between the behavioral and the neural level may appear intuitively plausible, it speaks against the implicit assumption of past investigations relating the value system to option-specific value comparisons. The current data should not be misinterpreted as implying that the neural reward system does not encode discounted subjective values at all; instead, they highlight the importance of individual variation in decision-making strategies, which influence how preferences are computed in the brain. Besides inter-individual differences, there is also evidence for intra-individual variation in the used decision strategy (i.e., an individual uses different value comparison strategies for different choice problems) as well as for the influence of contextual factors (e.g., time pressure leads to more attribute-wise comparisons)^16^. The neural reward system may, therefore, represent different utility functions for the same choice problem depending on such internal and external determinants.

The assumption that individuals generally compute the subjective values of rewards according to economic discount functions was already challenged by recent findings in the psychological literature on intertemporal decisions^15–18^, and we generalize these findings to further domains of decision-making like social preferences^8^. Still, these psychological accounts lacked biological plausibility, given that the dominant stream in the neural literature assumed that the neural reward system encodes subjective reward values, though without explicitly testing the alternative hypothesis that the brain might encode a different decision strategy. Our finding that activation in the reward system reflects the value comparison strategy employed by an individual provides evidence for the biological plausibility of these recent models of decision-making. By this, our findings also change our conceptual understanding of the neural basis of intertemporal decisions as they challenge the widely held assumption that the neural reward system encodes hyperbolically discounted subjective reward values^4,14,28^, and also past pharmacological studies on the role of dopamine on intertemporal choice relied on this assumption^25,30,31^. We note that we replicated previous findings that attribute-wise comparisons explain intertemporal choices better than option-wise comparisons^15,16^, whereas we observed no significant differences between value comparison strategies in the interpersonal decision task. Surprisingly, we also found that dopamine antagonism biased option-wise comparisons more strongly in the interpersonal than the intertemporal decision task. Decision makers may, therefore, employ dissociable value computation strategies for different types of cost-benefit decisions, and also, the role of the dopaminergic reward system may potentially vary between decision tasks. We show that it can be misleading to assume the reward system to encode the same value function in all individuals and contexts, given the influence of individual variation and contextual factors on the employed value computation strategy^15,16^.

From a broader perspective, our findings emphasize the importance of testing models of decision-making at the neural level. Studies assuming that the brain encodes discounted subjective reward values commonly did not explore whether alternative models of decision-making provide a better explanation for the observed brain activation. Past studies conducting such model comparisons, in turn, compared only decision models that were implicitly based on option-wise utility comparisons^33^. It is thus crucial to explore a broad range of behavioral models to prevent misinterpretation of neuroimaging findings, not only in the domain of decision-making. From this perspective, model comparisons at the neural level inform not only about the biological plausibility of competing models of human behavior but also advance our understanding of the precise computations implemented by a brain region.

## Methods

### Participants and ethics statement

#### fMRI study

A total of 35 volunteers took part in the fMRI experiment (mean age = 26.2 years; standard deviation = 2.74 years; 11 females, 24 males). All participants had normal or corrected-to-normal vision, were screened for counterindications of fMRI, and gave informed written consent prior to participation. The study was approved by the ethics committee of the psychology department at the Ludwig Maximilian University Munich (46_Soutschek_a). All ethical regulations relevant to human research participants were followed. Participants were compensated with 10 euro/hour plus a performance-dependent bonus (see below).

#### Dopamine antagonist study

56 volunteers (27 female, *M*_age_ = 23.2 years, SD_age_ = 3.1 years) received 400 mg amisulpride or placebo in two separate sessions (2 weeks apart) in a double-blind, randomized, within-subject crossover design^12^. Participants gave informed written consent before participation. The study was approved by the Cantonal Ethics Committee Zurich (2012-0568).

### Stimuli and task design

Participants performed three tasks in the fMRI scanner: an intertemporal decision task, an interpersonal decision task, and an environmental decision task. Experimental details and results for the environmental decision task will be reported in a separate manuscript. In the intertemporal decision task, participants chose between smaller-sooner and larger-later rewards (e.g., “3 euro in 0 days” versus “5 euro in 10 days”). The magnitude of the smaller reward varied from 0.5 to 5 euro (in steps of 0.5) and was delivered after delays ranging from 0 to 90 days, whereas the larger reward was fixed to 5 euro and delivered after 10–360 days. For the interpersonal decision task, participants first had to imagine a list of 100 people ranging from 0 to 100 (with 0 representing themselves, 1 the person socially closest to them, 50 a person that the participant had seen before without knowing the name, and 100 a random stranger on the street). In the task in the scanner, participants decided between assigning a variable amount of money (5.5–10 euro in steps of 0.5) to themselves or a close other (social distances 0, 10, or 20) or equally split the amount of 10 euro between the persons from the closer and the more distant options (e.g., “7.5 euro for themselves” versus “5 euro for both themselves and the person at social 20”). For the more distant option, we used the social distances of 1, 10, 20, 50, and 100.

In both tasks, participants had to indicate their choices via keypresses on an MR-compatible button box during the offer presentation for 5 s. After a decision, the chosen option turned red for the remaining stimulus presentation time. Trials were separated by jittered inter-trial intervals drawn from a Poisson distribution (mean = 3 s, minimum = 0.5 s). Within each run, the three tasks were administered in miniblocks of six trials, with the task order of the miniblocks being pseudo-randomized across each run. Participants performed a total of five runs, with one run including three miniblocks for each task. This resulted in a total of 90 trials for each task.

In the *dopamine antagonist study*, participants made intertemporal and interpersonal choices 90 min after amisulpride or placebo intake. In the intertemporal decision task, participants decided between a smaller-sooner reward (5–250 Swiss francs delivered after delays of 0–30 days) and a larger-later reward (15–300 Swiss francs delivered after 3–90 days). Participants made a total of 20 intertemporal decisions in an adaptive fashion where the choice options on each trial were designed such that the information provided by each decision was optimized (for details, see ref. ^12^). In the interpersonal decision task, participants made 54 choices between a selfish reward option (7.5–15.5 Swiss francs only for the participant) and sharing 15 Swiss francs equally between themselves and another person at social distance 1, 5, 10, 20, 50, or 100.

### Data acquisition

Images were acquired using a Siemens Magnetom Prisma 3 T scanner with a 64-channel head coil at the NeuroImaging Labor at the City Centre Campus of LMU Munich (NICUM). We acquired gradient echo T2*-weighted echo-planar images (EPIs) with blood-oxygen-level-dependent (BOLD) contrast (slices = 48; repetition time = 1 s). Participants completed five runs of the experiment in the scanner. Imaging parameters were the following: echo time = 30 ms; field of view = 240 mm, slice thickness = 3 mm, interslice gap = 0.3 mm. We also acquired a T1-weighted structural image (voxel size = 0.8 mm) for each participant. High-resolution structural scans were coregistered to their mean EPIs and averaged together to permit anatomical localization of the functional activations at the group level.

### Statistics and reproducibility

#### *Behavioral analysis*

Behavioral analyses were conducted in R. To test whether participants’ choices in the intertemporal and interpersonal decision tasks can better be explained by attribute-wise or option-wise comparisons, we fitted Bayesian drift-diffusion models (DDMs) using the JAGS software package^34^. JAGS utilizes Markov Chain Monte Carlo sampling for Bayesian estimation of drift-diffusion parameters (drift rate *ν*, boundary *α*, bias *ζ*, and non-decision time *τ*) via the Wiener module^35^. In our models, the upper decision boundary was associated with a choice of the larger-later or more distant option, and the lower boundary with a choice of the smaller-sooner or closer option. A positive drift rate thus indicated evidence accumulation towards the larger-later/distant option and a negative drift rate towards the smaller-sooner/closer option. In line with previous procedures^15^, we ran separate models where the drift rate reflected either attribute-wise (DDM_attribute_) or option-wise (DDM_option_) decision strategies. In attribute-wise comparisons, the drift rate v is given by the linear combination of the weighted comparisons of reward magnitudes and (temporal or social) distances between the choice options:

5 $${{{\rm{\nu }}}}={{{{\rm{\beta }}}}}_{{{{\rm{intercept}}}}}+{{{{\rm{\beta }}}}}_{{{{\rm{reward}}}}}\times {{{{\rm{Reward}}}}}_{{{{\rm{diff}}}}}+{{{{\rm{\beta }}}}}_{{{{\rm{distance}}}}}\times {{{{\rm{Distance}}}}}_{{{{\rm{diff}}}}}$$

where Reward_diff_ and Distance_diff_ indicate the difference in reward magnitudes and temporal or psychological distances, respectively, between the more distant and the closer options. *β*_reward_ and *β*_distance_ represent the individual weights assigned to differences in reward magnitude and distance, respectively, during evidence accumulation, whereas *β*_intercept_ is a participant-specific intercept. We note that DDM_attribute_ included no separate choice consistency parameter for the drift rate as this would make the model parameters unidentifiable, but in principle, Eq. (1) could be re-written as follows to include a separate choice consistency parameter^36^:

6 $${\nu }={{{{\rm{\beta }}}}}_{{{{\rm{intercept}}}}}+{{{{\rm{\beta }}}}}_{{{{\rm{reward}}}}} \times {{{{\rm{Reward}}}}}_{{{{\rm{diff}}}}}+{{{{\rm{\beta }}}}}_{{{{\rm{distance}}}}}\times {{{{\rm{Distance}}}}}_{{{{\rm{diff}}}}}$$

For option-wise comparisons, the drift rate was modeled to reflect comparisons of discounted subjective reward values:

7 $${\nu }^{\prime}={{{{\rm{\beta }}}}}_{{{{\rm{temperature}}}}} \times ({{{{\rm{SV}}}}}_{{{{\rm{distant}}}}}-{{{{\rm{SV}}}}}_{{{{\rm{close}}}}})$$

The hyperbolically discounted subjective reward values were given by

8 $${{{\rm{SV}}}}={{{\rm{reward}}}}\; {{{\rm{magnitude}}}}/(1+{{{{\rm{\beta }}}}}_{{{{\rm{discount}}}}} \times {{{\rm{distance}}}})$$

Here, *β*_discount_ represents the individual (log-transformed) discount factor in the intertemporal or interpersonal decision task. In both DDM_attribute_ and DDM_option_, we also modeled the starting bias *ζ*, the decision threshold *α*, and the non-decision time *τ*. Because previous studies suggest concave rather than linear relationships between drift rates and option-wise value comparisons, we transformed *v*’ with a sigmoidal link function (note that this also ensured that DDM_attribute_ and DDM_option_ entail the same number of parameters, whereas a model without this sigmoidal transformation provided a worse fit; see results section):

9 $${v}=2 \times {{v}}_{{{\mathrm{lim}}}}/(1+\exp (-{v}^{\prime}))-{v}_{{{\mathrm{lim}}}}$$

We estimated all parameters separately for each participant and task and calculated the widely applicable information criterion (WAIC) as a measure of model fit (with lower values indicating better model fit). The WAIC represents a generalized form of the Akaike information criterion (AIC) and can be computed without any information about the true probability distribution^37^. Unreasonable fast decision times (<250 ms; 6% of all trials in both tasks) were excluded from the analysis^21^. We note that the results of the behavioral model comparisons are robust to entering all trials in the analyses. We used flat uniform priors over reasonable parameter ranges (from −10 to +10 for the drift parameters and restricted to the positive range for the other parameters). For model estimation, we computed 2 chains with 10,000 samples (burning = 5000). $\hat{R}$ was used to assess model convergence in addition to visual inspection of chains. For all effects, $\hat{R}$ was below 1.01, indicating model convergence.

We conducted a model recovery analysis by re-computing DDM_attribute_ and DDM_option_ on data sets which were simulated based on the original parameters from DDM_attribute_ and DDM_option_. We checked whether the simulated data could best be explained by the model used for creating the data set.

We used the same modeling approach for behavioral data analysis in the fMRI and the dopamine antagonist study. In the dopamine study, we moreover fitted separate models for choices under amisulpride versus placebo. To analyze how amisulpride changed the propensity for attribute-wise relative to option-wise utility comparisons, we computed the difference between WAIC_attribute_–WAIC_option_ scores under amisulpride and WAIC_attribute_–WAIC_option_ scores under placebo (WAIC_diff_amisulpride_placebo_). The WAIC_diff_amisulpride_placebo_ difference scores were then regressed on predictors for Task (0 = interpersonal, 1 = intertemporal), Session (to control for task repetition effects; amisulpride administered in session 1 versus 2), WAIC_attribute_–WAIC_option_ scores under placebo as measure of the dominant decision strategy in the baseline condition (WAIC_diff_placebo_), and all interaction effects in a general linear model using the lme4 package in R^38^.

10 $${{{{\rm{WAIC}}}}}_{{{{\rm{diff}}}}\_{{{\rm{amisulpride}}}}\_{{{\rm{placebo}}}}} =	 \, {{{\rm{\beta }}}}1({{{\rm{Intercept}}}})+{{{{\rm{\beta }}}}}_{2}({{{\rm{Task}}}})+{{{{\rm{\beta }}}}}_{3}({{{\rm{Session}}}})+{{{{\rm{\beta }}}}}_{4}({{{{\rm{WAIC}}}}}_{{{{\rm{diff}}}}\_{{{\rm{placebo}}}}})\\ 	+{{{{\rm{\beta }}}}}_{5}({{{\rm{Task}}}} \times {{{\rm{Session}}}})+{{{{\rm{\beta }}}}}_{6}({{{\rm{Task}}}} \times {{{{\rm{WAIC}}}}}_{{{{\rm{diff}}}}\_{{{\rm{placebo}}}}})\\ 	+{{{{\rm{\beta }}}}}_{7}({{{\rm{Session}}}} \times {{{{\rm{WAIC}}}}}_{{{{\rm{diff}}}}\_{{{\rm{placebo}}}}})+{{{{\rm{\beta }}}}}_{8}({{{\rm{Task}}}} \times {{{\rm{Session}}} }\\ 	\, \times {{{{\rm{WAIC}}}}}_{{{{\rm{diff}}}}\_{{{\rm{placebo}}}}})$$

Because the dependent variable measures the strength of the amisulpride effect on participants’ trade-off between attribute-wise and option-wise processing, a significant effect of WAIC_diff_placebo_ indicates that the amisulpride effect depends on the dominant value comparison strategy (i.e., WAIC_attribute_–WAIC_option_) under placebo. All predictors were *z*-transformed in order to avoid the issue of arbitrarily defining one-factor level as a reference category (which distorts the interpretation of main effects and lower-level interaction terms). Degrees of freedom were estimated via the Satterthwaite approximation.

#### Imaging analysis

Analysis of neuroimaging data was performed with SPM12 in Matlab (www.fil.ion.ucl.ac.uk/spm). The functional images of each participant were motion corrected, unwarped, slice-timing corrected (temporally corrected to the first image), and co-registered to the anatomical image. Following segmentation, we spatially normalized the data into standard MNI space. Finally, data were smoothed with a 6 mm FWHM Gaussian kernel and high-pass filtered (filter cutoff = 128 seconds).

For the first-level analysis of the imaging data, we conducted separate general linear models (GLMs) to determine the neural correlates of utility representations according to either attribute-wise (GLM_attribute_) or option-wise (GLM_option_) comparisons. GLM_attribute_ included separate onset regressors for the intertemporal and interpersonal decision tasks (modeled for the duration of the decision screen presentation), which were modulated by parametric modulators for the z-transformed absolute value of the trial-wise drift rate as a measure of participants’ preference strength for one option over the other. Trial-wise drift rates were given via the individually estimated parameters from DDM_attribute_. GLM_option_ was identical with GLM_attribute_ with the only difference being that trial-wise drift rates were provided by DDM_option_ rather than DDM_attribute_. In all models, we added onset regressors for the environmental decision task and for response omission trials, as well as 6 movements (3 translation and 3 rotation) parameters as covariates of no interest. We convolved regressors with the canonical hemodynamic response function in SPM.

For statistical analysis, we first computed participant-specific contrasts for utility representations in GLM_attribute_ and GLM_option_, separately for the intertemporal and interpersonal decision task. For the second-level analysis, we entered the contrast images from all participants into a between-participant, random effects analysis and conducted whole-brain second-level analyses using one-sample *t-*tests. For these analyses, we reported results that survived whole-brain family-wise error (FWE) corrections at the peak or cluster level (*p* < 0.05). For the figures, we set the individual voxel threshold to *p* < 0.001 with a minimal cluster extent of *k* ≥ 20 voxels. We reported results using the MNI coordinate system. To test the apriori hypothesis that the neural reward system represents both attribute-wise and option-wise value comparisons, we extracted parameter estimates from two regions of interest (ROIs) using the Marsbar toolbox^39^. These ROIs were defined based on a meta-analysis of the neural basis of value coding^19^ and comprised spheres with a diameter of 6 mm (length of spatial smoothing filter) around the peak coordinates in the striatum (*x* = −3, *y* = 10, *z* = −4) and the VMPFC (*x* = −1, *y* = 46, *z* = −7). We used these ROIs to extract parameter estimates and submitted the extracted values to one-sample *t*-tests, Bonferroni-correcting for the number of pre-defined ROIs (corrected threshold: 5%/2 = 2.5%).

Finally, we tested whether neural activation in the value system is better explained by attribute-wise than by option-wise utility comparisons. For this purpose, we first computed two further GLMs, one where the onset regressor for the intertemporal decision task was moderated by trial-varying drift rates from DDM_attribute_ and the onset regressor for the interpersonal decision task by drift rates based on DDM_option_ (GLM_option-interpersonal_). Likewise, in a further model the onset regressor for the intertemporal decision task was moderated by trial-varying drift rates from DDM_option_ and the onset regressor for the interpersonal decision task by drift rates based on DDM_attribute_ (GLM_option-intertemporal_). These additional GLMs allowed us to compare model fits separately for the intertemporal (GLM_attrbute_ versus GLM_option-intertemporal_) and the interpersonal decision task (GLM_attrbute_ versus GLM_option-interpersonal_). We then used the MACS toolbox^29^ to obtain participant-specific statistical maps of the goodness of fit (using the Bayesian information criterion (BIC)) of GLM_attribute_, GLM_option-intertemporal_, and GLM_option-interpersonal_ in each voxel. We then entered the differences between the BICs (GLM_attribute_ minus GLM_option-intertemporal_ and GLM_attribute_ minus GLM_option-interpersonal_) into a second-level analysis. To test the hypothesis that the striatum more strongly represents attribute-wise than option-wise value comparisons, we extracted BIC differences from our ROIs using the Marsbar toolbox^39^ and submitted the extracted BIC values to one-sample *t*-tests.

### Statistics and reproducibility

For the reported behavioral analyses, we used R (version 4.0.0); for the imaging analysis, we used the toolbox SPM 12 within Matlab R2019b. All figures were created using Matlab 2019b. As robustness checks and to improve the reproducibility of our findings, we compared two different domains of cost-benefit decisions and used different value coding ROIs in the imaging analysis.

### Reporting summary

Further information on research design is available in the Nature Portfolio Reporting Summary linked to this article.

## Supplementary information

Supplementary Information

Description of Additional Supplementary File

Supplementary Data

Reporting Summary

## Data Availability

The behavioral data supporting the findings of this study and the data analysis code are available on Open Science Framework (https://osf.io/krxe4/?view_only=48fe85361aea4c75a18d44b41765e227)^40^. Imaging data are provided by the authors upon reasonable request. Numerical source data for all graphs in the manuscript can be found in the Supplementary Data file.
