# Supplementary material for: Neural reward system reflects individual value comparison strategy in cost-benefit decisions
Source: Commun Biol. 2024 Nov 12;7:1488. doi: 10.1038/s42003-024-07210-5 (PMC11557971; doi:10.1038/s42003-024-07210-5)
Supplement: Supplementary file 1 — Supplementary Information [file 42003_2024_7210_MOESM1_ESM.pdf]

Supplementary Information for

Neural reward system reflects individual value comparison strategy in cost-benefit decisions

Zarah Le Houcq Corbi and Alexander Soutschek

Department for Psychology, Ludwig Maximilian University Munich, Munich, Germany

Tables

Table S1. Anatomical locations and MNI coordinates of the peak activations correlating with absolute drift rates (as measure of preference strength) in GLM_{option}. We report activations surviving whole-brain FWE correction at peak or cluster level ($p < 0.05$).

Hem = Hemisphere (L = left, R = right); BA = Brodmann area

			MNI Coordinates				
Region	Hem	BA	X	Y	Z	k	T
Caudate	R/L		-4	15	15	431	5.33
Cerebellum	L		-31	-42	-93	97	5.19

Table S2. Anatomical locations and MNI coordinates of the peak activations correlating with absolute drift rates (as measure of preference strength) in $GLM_{attribute}$. We report activations surviving whole-brain FWE correction at peak or cluster level ($p < 0.05$).

Hem = Hemisphere (L = left, R = right); BA = Brodmann area

			MNI Coordinates				
Region	Hem	BA	X	Y	Z	k	T
Cerebellum	L		-34	-39	-3	180	5.68
Caudate	R/L		2	12	-9	79	5.20

Table S3. Influence of amisulpride versus placebo on the balance between attribute-wise and option-wise value comparisons. We regressed differences in mode fit ($WAIC_{attribute}$ minus $WAIC_{option}$) between amisulpride and placebo on predictors for Task (intertemporal versus interpersonal), Session (amisulpride administered in session 1 versus 2), and the dominant value computation strategy under placebo (WAICdiff_placebo = $WAIC_{attribute}$ minus $WAIC_{option}$ placebo). We report standard errors of the mean in brackets.

**Regressor**	**beta**	**t**	**df**	**p**
Intercept	1.78 (1.32)	1.35	82	0.18
Task	3.90 (1.30)	2.99	81	0.004
Session	-0.86 (1.32)	0.65	82	0.52
$WAIC_{diff\_placebo}$	-9.61 (1.57)	6.11	104	<0.001
Task × Session	-1.25 (1.31)	0.95	81	0.34
Task × $WAIC_{diff\_placebo}$	1.01 (1.57)	0.64	102	0.52
Session × $WAIC_{diff\_placebo}$	1.03 (1.58)	-0.65	104	0.51
Task × Session × $WAIC_{diff\_placebo}$	0.21 (1.58)	0.13	102	0.90
