# Supplementary material for: Neural reward system reflects individual value comparison strategy in cost-benefit decisions
Source: Commun Biol. 2024 Nov 12;7:1488. doi: 10.1038/s42003-024-07210-5 (PMC11557971; doi:10.1038/s42003-024-07210-5)
Supplement: Supplementary file 2 — Description of Additional Supplementary File [file 42003_2024_7210_MOESM2_ESM.pdf]

Description of additional supplementary file

File name: supplementary data

Description: source data for figures
