# Supplementary material for: Neural reward system reflects individual value comparison strategy in cost-benefit decisions
Source: Commun Biol. 2024 Nov 12;7:1488. doi: 10.1038/s42003-024-07210-5 (PMC11557971; doi:10.1038/s42003-024-07210-5)
Supplement: Supplementary file 4 — Reporting Summary [file 42003_2024_7210_MOESM4_ESM.pdf]

nature portfolio

Corresponding author(s): Alexander Soutschek

Last updated by author(s): May 15, 2024

Reporting Summary

Nature Portfolio wishes to improve the reproducibility of the work that we publish. This form provides structure for consistency and transparency in reporting. For further information on Nature Portfolio policies, see our Editorial Policies and the Editorial Policy Checklist.

Statistics

For all statistical analyses, confirm that the following items are present in the figure legend, table legend, main text, or Methods section.

n/a	Confirmed	
☐	☒	The exact sample size (*n*) for each experimental group/condition, given as a discrete number and unit of measurement
☐	☒	A statement on whether measurements were taken from distinct samples or whether the same sample was measured repeatedly
☐	☒	The statistical test(s) used AND whether they are one- or two-sided<br>*Only common tests should be described solely by name; describe more complex techniques in the Methods section.*
☐	☒	A description of all covariates tested
☐	☒	A description of any assumptions or corrections, such as tests of normality and adjustment for multiple comparisons
☐	☒	A full description of the statistical parameters including central tendency (e.g. means) or other basic estimates (e.g. regression coefficient) AND variation (e.g. standard deviation) or associated estimates of uncertainty (e.g. confidence intervals)
☐	☒	For null hypothesis testing, the test statistic (e.g. *F*, *t*, *r*) with confidence intervals, effect sizes, degrees of freedom and *P* value noted<br>*Give P values as exact values whenever suitable.*
☐	☒	For Bayesian analysis, information on the choice of priors and Markov chain Monte Carlo settings
☐	☒	For hierarchical and complex designs, identification of the appropriate level for tests and full reporting of outcomes
☐	☒	Estimates of effect sizes (e.g. Cohen's *d*, Pearson's *r*), indicating how they were calculated

Our web collection on statistics for biologists contains articles on many of the points above.

Software and code

Policy information about availability of computer code

Data collection	Matlab 2019, Cogent toolbox
Data analysis	Matlab 2019, R 4.0.0

For manuscripts utilizing custom algorithms or software that are central to the research but not yet described in published literature, software must be made available to editors and reviewers. We strongly encourage code deposition in a community repository (e.g. GitHub). See the Nature Portfolio guidelines for submitting code & software for further information.

Data

Policy information about availability of data

All manuscripts must include a data availability statement. This statement should provide the following information, where applicable:

- Accession codes, unique identifiers, or web links for publicly available datasets
- A description of any restrictions on data availability
- For clinical datasets or third party data, please ensure that the statement adheres to our policy

The data that support the findings of this study are available on Open Science Framework (https://osf.io/krxe4/?view_only=48fe85361aea4c75a18d44b41765e227).

Human research participants

Policy information about studies involving human research participants and Sex and Gender in Research.

Reporting on sex and gender	We collected balanced samples of female and male participants. We had no apriori hypothesis regarding gender differences, therefore we did not statistically test for gender differences.
Population characteristics	We collected healthy young participants (age: 18-35 years) from the student populations in Munich and Zurich.
Recruitment	We recruited via internal participant pools at the Universities of Munich and Zurich.
Ethics oversight	The fMRI study was approved by the ethics committee of the psychology department at the University of Munich. The pharma study was approved by the Cantonal ethics committee Zurich.

Note that full information on the approval of the study protocol must also be provided in the manuscript.

Field-specific reporting

Please select the one below that is the best fit for your research. If you are not sure, read the appropriate sections before making your selection.

☐ Life sciences ☒ Behavioural & social sciences ☐ Ecological, evolutionary & environmental sciences

For a reference copy of the document with all sections, see nature.com/documents/nr-reporting-summary-flat.pdf

Behavioural & social sciences study design

All studies must disclose on these points even when the disclosure is negative.

Study description	The fMRI study followed a within-subject design where in one experimental session participants performed decision tasks in the MRI scanner.<br>The dopamine study followed a within-subject design where all participants received the D2 antagonist amisulpride or placebo (on different testing days) before performing a computer-based decision making tasks.
Research sample	For the fMRI study, we recruited 35 participants from the student population in Munich. For the dopamine study, we recruited 56 participants from the student population in Zurich. As is common practice in neuroscience research, the sample was not representative of the entire population, however we investigated basic neuro-cognitive processes and were not interested in inter-individual variation.
Sampling strategy	No apriori power analysis was conducted for the fMRI study but we aimed for a sample size that is larger than in most previous imaging studies on intertemporal and social decision making.<br>The reported analyses of the dopamine study are based on a re-analysis of an existing data set, no power analysis for the current analyses was conducted.
Data collection	Participants performed computer-based tasks either in the MRI scanner (fMRI experiment) or after receiving amisulpride or placebo in the dopamine study. Participants and experimentors were blind to the current drug condition (double-blind design) in the dopamine study.
Timing	Data for the fMRI study were collected in summer and autumn 2022.<br>Data for the dopamine study were collected in 2016 (February-November).
Data exclusions	No data were excluded from the analyses.
Non-participation	No volunteers declined participation.
Randomization	Participants in the dopamine study were randomly assigned to receiving either placebo or amisulpride in the first experimental session.

Reporting for specific materials, systems and methods

We require information from authors about some types of materials, experimental systems and methods used in many studies. Here, indicate whether each material, system or method listed is relevant to your study. If you are not sure if a list item applies to your research, read the appropriate section before selecting a response.

Materials & experimental systems

n/a	Involved in the study
☒	☐ Antibodies
☒	☐ Eukaryotic cell lines
☒	☐ Palaeontology and archaeology
☒	☐ Animals and other organisms
☒	☐ Clinical data
☒	☐ Dual use research of concern

Methods

n/a	Involved in the study
☒	☐ ChIP-seq
☒	☐ Flow cytometry
☐	☒ MRI-based neuroimaging

Magnetic resonance imaging

Experimental design

Design type: event-related

Design specifications: 5 runs per session, a total of 90 trials per task

Behavioral performance measures: binary choice data, decision times

Acquisition

Imaging type(s): functional

Field strength: 3 T

Sequence & imaging parameters: We acquired gradient echo T2*-weighted echo-planar images (EPIs) with blood-oxygen-level-dependent (BOLD) contrast (slices = 48; repetition time = 1 s). Participants completed five runs of the experiment in the scanner. Imaging parameters were the following: echo time = 30 ms; field of view = 240 mm, slice thickness = 3 mm, interslice gap = 0.3 mm.

Area of acquisition: whole brain

Diffusion MRI ☐ Used ☒ Not used

Preprocessing

Preprocessing software: SPM 12

Normalization: standard routines in SPM 12

Normalization template: MNI template

Noise and artifact removal: temporal filter of 128 s (default in SPM 12)

Volume censoring: Not performed

Statistical modeling & inference

Model type and settings: univariate with first-level and random effect second-level models

Effect(s) tested: neural correlates of drift rates

Specify type of analysis: ☐ Whole brain ☐ ROI-based ☒ Both

Anatomical location(s): ROIs were based on meta-analysis on value coding

Statistic type for inference (See Eklund et al. 2016): cluster-wise FWE correction (cluster.defining threshold of p < 0.001 uncorrect)

Correction: FWE

Models & analysis

n/a	Involved in the study
☒	☐ Functional and/or effective connectivity
☒	☐ Graph analysis
☒	☐ Multivariate modeling or predictive analysis
